# Isoliquiritigenin Inhibits Triple-Negative Breast Cancer Progression via Targeting the IRF5/SLC7A5/IDO1-Mediated Tryptophan Metabolism Pathway

**DOI:** 10.32604/or.2025.068292

**Published:** 2025-10-22

**Authors:** Sihai Duan, Xiaoyan Li, Cailu Song, Song Wu, Yunyun Tang, Qing Bao, Na Li, Hailin Tang

**Affiliations:** 1 Department of Breast and Thyroid Surgery, The Central Hospital of Yongzhou, Yongzhou, 425007, China; 2 Department of Breast and Thyroid Surgery, Yongzhou Hospital Affiliated to University of South China, Yongzhou, 425007, China; 3 State Key Laboratory of Oncology in South China, Guangdong Provincial Clinical Research Center for Cancer, Sun Yat-sen University Cancer Center, Guangzhou, 510060, China; 4 Department of Basic Medicine, School of Health and Wellness Sciences, Guangzhou Kangda Vocational Technical College, Guangzhou, 510555, China

**Keywords:** Interferon regulatory factor 5 (IRF5), triple-negative breast cancer, tryptophan metabolism, isoliquiritigenin, solute carrier family 7 member 5 (SLC7A5), indoleamine 2,3-dioxygenase 1 (IDO1)

## Abstract

Objectives: Triple-negative breast cancer (TNBC) is the breast cancer subtype with the poorest prognosis. This study aimed to elucidate the molecular pathways through which isoliquiritigenin (ISL), a natural chalcone compound derived from licorice and other plant roots, targets interferon regulatory factor 5 (IRF5) in TNBC. Methods: TNBC cell lines were cultured and subjected to IRF5 knockdown using short hairpin RNA. Cell proliferation was assessed by cell counting kit-8 (CCK-8) assay and colony formation assays. Western blotting and quantitative reverse transcription polymerase chain reaction (RT-PCR) were employed to measure expression levels of IRF5, solute carrier family 7 member 5 (SLC7A5), and indoleamine 2,3-dioxygenase 1 (IDO1). Intracellular tryptophan and its metabolites were quantified using commercially available assay kits and high-performance liquid chromatography (HPLC). TNBC cells were treated with various concentrations of ISL to evaluate its effects on proliferation and tryptophan metabolism. Results: IRF5 was highly expressed in TNBC cell lines. Silencing IRF5 significantly inhibited cellular proliferation and growth. Knockdown of IRF5 reduced the expression of SLC7A5 and IDO1, leading to decreased intracellular levels of tryptophan and its metabolites. ISL markedly suppressed TNBC cell proliferation and disrupted tryptophan metabolism in tumor cells. Conclusion: ISL may inhibit TNBC progression by downregulating IRF5 and interfering with SLC7A5/IDO1-mediated tryptophan metabolic reprogramming, suggesting a potential therapeutic mechanism for TNBC treatment.

## 1 Introduction

Breast cancer ranks among the most common malignant neoplasms globally, ranking second only to lung cancer [1]. The death toll significantly exceeds that of other gynecological malignancies such as uterine and ovarian cancers [1]. Triple-negative breast cancer (TNBC) represents one of the most therapeutically challenging subtypes of breast cancer, characterized by high heterogeneity, pronounced invasiveness, and metastatic potential, as well as a lack of well-defined therapeutic targets [2]. Currently, TNBC accounts for approximately 20% of global breast cancer cases [3]. Studies indicate that TNBC has a high risk of distant metastasis within 3 to 5 years after initial treatment [4]. Even though the prevalence of TNBC is comparatively low, this subtype of breast cancer is associated with roughly 83% of all fatalities related to breast cancer [4,5]. TNBC exhibits limited responsiveness to conventional targeted therapies, and its key molecular drivers are not well understood. Therefore, chemotherapy remains the mainstay of treatment [6]. Despite TNBC’s relatively high sensitivity to chemotherapy, it exhibits a high rate of recurrence and substantially reduced overall survival (OS) in comparison with other breast cancer subtypes [7]. Therefore, unraveling the molecular mechanisms underpinning the progression of TNBC and identifying novel therapeutic targets represent urgent challenges in current clinical research.

Isoliquiritigenin (ISL) is a natural chalcone compound derived from licorice root, widely recognized for its diverse pharmacological properties, such as anti-inflammatory, antioxidant, and anticancer effects [8,9]. Increasing evidence has demonstrated that ISL exerts potent inhibitory effects on the proliferation, migration, and invasion of various cancer types, including breast cancer [10–12]. For example, ISL has been reported to effectively inhibit brain metastasis of TNBC by modulating the circNAV3/miR-4262/ST6GALNAC5/EGFR axis, thereby suppressing tumor cells’ ability to penetrate the blood-brain barrier and impeding metastatic progression [13]. Notwithstanding these promising results, the specific molecular pathways by which ISL mediates its antitumor effects in TNBC have not been fully elucidated. In particular, the role of ISL in regulating tumor metabolism, especially tryptophan metabolism, which is critically involved in tumor immune evasion and progression, is yet to be fully elucidated [14,15].

The interferon regulatory factor (IRF) family comprises transcription factors that play a central role in regulating the expression of interferon (IFN) genes [16,17]. The IRF5 gene, located on chromosome 7q32, encodes the IRF5 protein [18]. The IRF5 protein contains nuclear localization signals (NLS) at both its N- and C-C-termini, which, together with nuclear export signals (NES), are critical for its transport between the cytoplasm and nucleus [19]. In recent years, IRF5 has emerged as a focus of tumor research. Pimenta and colleagues reported that loss of IRF5 in breast cancer cells leads to dysregulation of cytokines and chemokines, subsequently impairing immune cell recruitment to tumor sites [20]. Although IRF5 was initially considered a tumor suppressor and identified as a direct target of p53 [21]. Studies demonstrate that its ability to induce genes involved in apoptosis and cell cycle regulation is independent of p53, suggesting that IRF5 and p53 exert tumor-suppressive effects via distinct pathways [22]. Despite its tumor-suppressive activities, numerous studies have also revealed oncogenic roles of IRF5: Fang et al. found that IRF5 promotes glycolysis and progression of hepatocellular carcinoma [23]. Besides, a study showed that cytoplasmic IRF5 facilitates the degradation of Wnt5a and E-cadherin, thereby promoting metastasis in gastric cancer cells [24]. However, the precise mechanisms underlying IRF5’s role in triple-negative breast cancer remain unclear and warrant further investigation.

This research intends to assess the function of ISL in tryptophan metabolism and its effects on the proliferation and growth of TNBC cells. The results demonstrate that ISL exerts a significant inhibitory effect on the proliferation of TNBC cells *in vitro*, potentially by downregulating the IRF5/solute carrier family 7 member 5 (SLC7A5)/indoleamine 2,3-dioxygenase 1 (IDO1) pathway, thereby suppressing tryptophan metabolism in TNBC.

## 2 Methods and Materials

### 2.1 Cell Culture and Treatment

Human normal breast epithelial cells (MCF-10A) and a panel of breast cancer cell lines were obtained from the American Type Culture Collection (ATCC; Manassas, VA, USA). The breast cancer cell lines included T47D (a Luminal B subtype cell line), SK-BR-3 (a HER2-overexpressing subtype cell line), and several TNBC cell lines, namely MDA-MB-231, HCC1806, SUM159PT, BT-549, and MDA-MB-468. Prior to utilization, all cell lines underwent authentication via short tandem repeat (STR) profiling and were verified to be devoid of mycoplasma contamination. These cell lines were maintained in DMEM (11965118; Gibco, Grand Island, NY, USA) under standard conditions at 37°C with 5% CO_2_. Stable knockdown or overexpression of IRF5 in SUM159PT, BT-549, and MDA-MB-468 cells was achieved using shRNA or plasmids targeting IRF5 procured from Umine Biotechnology Co., Ltd. (Guangzhou, China). Isoliquiritigenin (ISL) was purchased from Sigma-Aldrich (I3766-10MG; Shanghai, China). A 100 mM stock solution was prepared in dimethyl sulfoxide (DMSO) and subsequently diluted to the desired concentrations (0, 6.25, 12.5, 25, 50, and 100 μM) in culture medium immediately before use.

### 2.2 Clinical Samples

A total of four Breast cancer tissue specimens were collected from patients undergoing surgery at Sun Yat-sen University Cancer Center (SYSUCC). Immediately after excision, samples were preserved in RNAlater solution to stabilize RNA integrity. Informed consent was obtained from all participants, and the study protocol received approval from the institutional ethics committee (GZKJ020-018). Tissue samples were stored at −80°C until further analysis. The investigations were conducted in accordance with ethical guidelines governing human subjects research.

### 2.3 Immunohistochemical (IHC) Staining

Antigen retrieval was performed using EDTA citrate buffer (ZLI-9069; ZSGB-BIO, Beijing, China) with heating in a pressure cooker. The stained sections were obtained from the previously collected Luminal A (Lum A), Luminal B (Lum B), Her2-positive (Her2+), and TNBC tissue samples. Following blocking, the sections were incubated with the primary antibody at 37°C for 1 h, followed by incubation with the secondary antibody for 30 min (PV-6000; ZSGB-BIO, Beijing, China). After washing with PBST, DAB staining was carried out (ZLI-9017; ZSGB-BIO, Beijing, China) and nuclei were counterstained with hematoxylin (G1120; Solarbio, Beijing, China).

### 2.4 Cell Counting Kit-8 Assay

Cell growth was quantified using the Cell Counting Kit-8 (C6005XL; UElandy, Suzhou, China). BT-549 and MDA-MB-468 cells were seeded into 96-well plates (2500/cells per well) and subjected to various experimental treatments for predetermined durations. At 24, 48, and 72 h, CCK-8 solution was diluted with culture medium at a ratio of 1:10, and 100 μL of the prepared solution was added to each well, followed by incubation in a cell culture incubator, absorbance values at 450 nm were recorded using a microplate reader (EPOCH2; BioTek, Winooski, VT, USA). All assays were conducted in triplicate to ensure reproducibility.

### 2.5 Colony Formation Assay

The colony formation assay was conducted by seeding BT-549 and MDA-MB-468 cells at a density of 500 cells/mL into culture dishes. The cells were then incubated under standard cell culture conditions (37°C, 5% CO_2_) for 10 to 14 days, allowing individual cells to proliferate and form discrete colonies. After the incubation period, the culture medium was carefully removed. The cells were gently washed twice with phosphate-buffered saline (PBS, 1.19 g/cm^3^, pH 7.4), fixed with methanol (100%) for 15 min, and stained with 0.1% crystal violet solution for 20 min. Colonies were observed and counted using a ChemiDoc imaging system (1708370; Bio-Rad, Hercules, CA, USA) and ImageJ (Version 2.0.0; Washington, DC, USA).

### 2.6 Quantitative Reverse Transcription Polymerase Chain Reaction

Total RNA was extracted from the above-mentioned breast cancer cell lines and normal breast epithelial cell lines using TRIzol reagent (15596026; Invitrogen, Carlsbad, CA, USA). cDNA was synthesized through reverse transcription using a kit from Takara (Tokyo, Japan). Quantitative real-time PCR was conducted employing SYBR Green Master Mix (Roche, Switzerland) on a Bio-Rad detection platform. The primers for IRF5 and GAPDH were purchased from Ruibiotech (Guangzhou, China). (IRF5-F: GGGCTTCAATGGGTCAACG; IRF5-R: GCCTTCGGTGTATTTCCCTG; GAPDH-F: TGTGGGCATCAATGGATTTGG; GAPDH-R: ACACCATGTATTCCGGGTCAAT)

### 2.7 Western Blotting

BT-549 and MDA-MB-468 cells lysates were prepared with RIPA buffer (Beyotime, Shanghai, China). Protein samples of equal amounts (20–30 μg) were resolved by SDS-PAGE and subsequently transferred to PVDF membranes (Millipore, Billerica, MA, USA). The membranes were blocked and then incubated overnight at 4°C with primary antibodies targeting GAPDH (#2118), SLC7A5 (#32683), and IRF5 (#E7F9W), all obtained from Cell Signaling Technology (Danvers, MA, USA), each diluted at a ratio of 1:1000, and IDO1 (#13268-1-AP; Proteintech, Wuhan, China) diluted at 1:1000. Protein bands were detected via enhanced chemiluminescence (ECL) reagents (3214863A; Thermo Fisher, Rockford, IL, USA) and visualized using a ChemiDoc imaging system (1708370; Bio-Rad, Hercules, CA, USA). GAPDH served as the loading control in all experiments.

### 2.8 Tryptophan and Kynurenine Quantification

BT-549 and MDA-MB-468 cells were cultured under appropriate conditions until reaching the desired density, collected, and washed with PBS (1.19 g/cm^3^, pH 7.4). According to the instructions of the tryptophan ELISA kit (abs580221; Absin, Shanghai, China) and KYN ELISA Kit (XG-EC1292; SigBio, Shanghai, China), samples and standards were added to a 96-well plate pre-coated with specific antibodies. Absorbance was measured at 450 nm using a microplate reader, and tryptophan concentrations in the samples were quantified based on the standard curve, thereby reflecting intracellular tryptophan metabolism.

### 2.9 High-Performance Liquid Chromatography

To determine the concentrations of different amino acids, BT-549 and MDA-MB-468 cells were rinsed twice with PBS (1.19 g/cm^3^, pH 7.4) and then lysed using an extraction solution composed of 80% methanol in water at −80°C for 30 min. The resulting lysates were centrifuged at 12,000× *g* for 10 min at 4°C, and the supernatant was harvested. This supernatant was processed through a series of steps, including extraction, filtration, drying, and reconstitution, before being analyzed using high-performance liquid chromatography coupled with mass spectrometry (HPLC-MS) (Thermo Ultimate 3000; Thermo Fisher, Rockford, IL, USA).

### 2.10 Gene Expression, Functional Enrichment, and Target Prediction Analysis

The expression differences of IRF5, IDO1, and SLC7A5 in breast cancer were analyzed using the UALCAN (https://ualcan.path.uab.edu/) portal based on the Cancer Genome Atlas (TCGA) database (https://www.genome.gov/Funded-Programs-Projects/Cancer-Genome-Atlas, accessed on 12 August 2025) [25]. Differentially expressed genes (DEGs) were subjected to network pathway and cluster analysis using Metascape (https://metascape.org/gp/index.html#/main/step1, accessed on 12 August 2025) [26]. Volcano plots illustrating DEGs between high and low IRF5 expression groups were generated using SRplot (http://www.bioinformatics.com.cn/srplot) [27]. Additionally, potential target genes of IRF5 were predicted using the JASPAR database (https://jaspar.elixir.no/) [28].

### 2.11 Statistical Analysis

All experiments were conducted in triplicate or more, and the data were analyzed using GraphPad Prism 10 (Version 10.3.1; San Diego, CA, USA). Results are expressed as mean ± standard deviation (SD). Differences between the two groups were evaluated using Student’s *t*-test. For multi-group data analysis, one-way ANOVA was performed to assess pairwise differences. The half-maximal inhibitory concentration (IC_50_) of ISL in MDA-MB-468 and BT-549 cell lines was calculated by fitting dose–response curves using nonlinear regression analysis in GraphPad Prism 10. Kaplan-Meier survival analysis of TNBC patients’ clinical data was also conducted using GraphPad Prism 10. A *p* < 0.05 was considered statistically significant.

## 3 Results

### 3.1 IRF5 as a Potential Driver Gene in Triple-Negative Breast Cancer Progression

**Figure 1:**
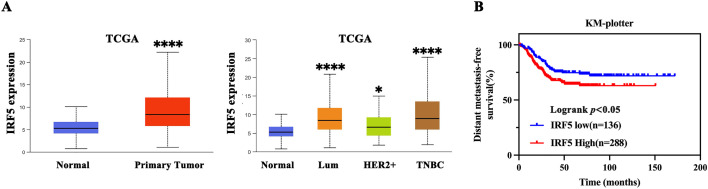

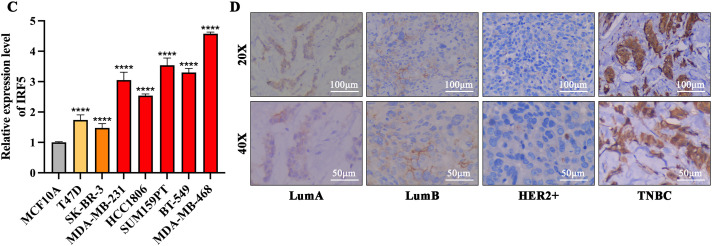
IRF5 as a Potential Driver Gene in Triple-Negative Breast Cancer Progression. (**A**) Comparison of IRF5 mRNA expression levels among various breast cancer molecular subtypes. (**B**) Kaplan-Meier survival analysis demonstrating a significantly shorter distant metastasis-free survival (DMFS) in breast cancer patients exhibiting high IRF5 expression. (**C**) qRT-PCR analysis of IRF5 expression in normal mammary epithelial cells and various breast cancer cell lines. (**D**) Immunohistochemical staining of breast cancer tissue samples under microscopy reveals markedly elevated IRF5 expression. *p < 0.05, ****p < 0.0001

Analysis of data from The Cancer Genome Atlas (TCGA) showed that IRF5 expression was significantly elevated in breast cancer tissues relative to normal breast tissue (Fig. 1A). Moreover, IRF5 expression was markedly higher in TNBC compared to Luminal and HER2-positive subtypes, indicating a strong correlation between IRF5 upregulation and TNBC progression (Fig. 1A). Kaplan-Meier survival analysis of clinical data from 424 surgically treated TNBC patients demonstrated that high IRF5 expression was associated with increased risk of distant metastasis post-surgery, highlighting IRF5’s potential role as a key promoter of TNBC progression (Fig. 1B). Then, we analyzed IRF5 expression across various breast cancer cell lines, with a focus on TNBC lines, using qRT-PCR. Compared to normal mammary epithelial MCF10A cells and other breast cancer subtypes, IRF5 expression was markedly upregulated in TNBC cell lines (Fig. 1C). Immunohistochemical analysis of breast cancer samples further confirmed pronounced IRF5 overexpression in all TNBC tumor tissues (Fig. 1D). Together, these findings suggest that IRF5 is a critical molecular driver of TNBC progression.

### 3.2 Inhibition of IRF5 Expression Significantly Suppresses Proliferation and Colony Formation in TNBC

**Figure 2:**
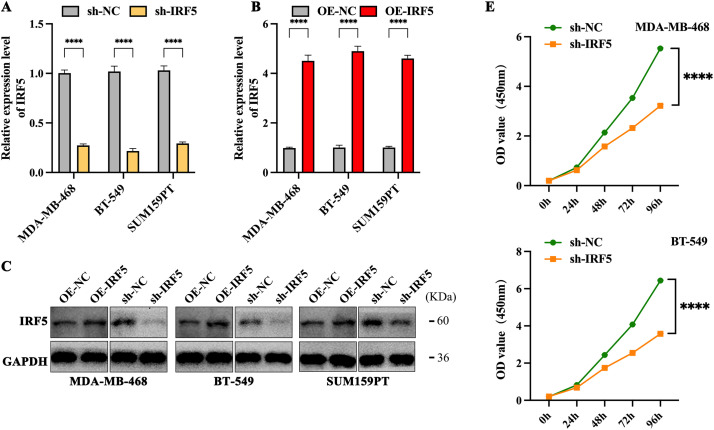

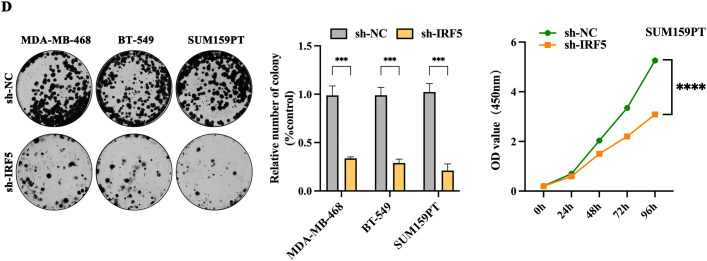
Inhibition of IRF5 expression significantly suppresses proliferation and colony formation in TNBC cells. (**A**) Validation of stable IRF5 knockdown efficiency in MDA-MB-468, BT-549, and SUM159PT cell lines by qRT-PCR. (**B**) Construction and qRT-PCR validation of TNBC cell lines stably overexpressing IRF5. (**C**) Western blot analysis confirming IRF5 overexpression and knockdown efficiencies in the indicated cell lines. (**D**,**E**) Colony formation (**D**) and CCK-8 proliferation assays (**E**) showing the suppressive effects of IRF5 knockdown on TNBC cell growth. ***p < 0.001, ****p < 0.0001

We selected three TNBC cell lines with the highest IRF5 expression—MDA-MB-468, BT-549, and SUM159PT—for subsequent experiments (Fig. 2A). IRF5 was either overexpressed or knocked down in these cell lines via viral vector transfection, followed by puromycin selection to establish stable cell lines with altered IRF5 expression. The efficacy of these modifications was confirmed by qRT-PCR and Western blot analyses (Fig. 2B,C). Colony formation assays demonstrated that IRF5 knockdown significantly reduced the number of colonies formed by TNBC cells (*p* < 0.001) (Fig. 2D). Consistently, CCK-8 proliferation assays showed that IRF5 knockdown markedly inhibited the proliferative capacity of TNBC cell lines (*p* < 0.0001) (Fig. 2E).

### 3.3 IRF5 Promotes Tryptophan Metabolism in TNBC

**Figure 3:**
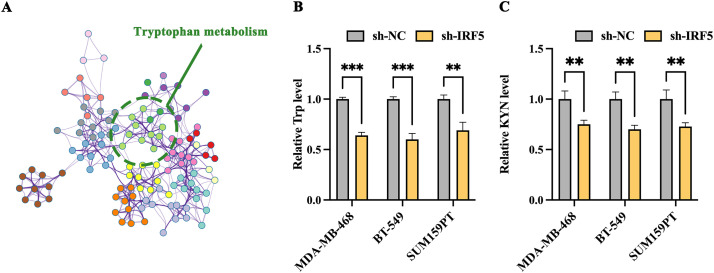

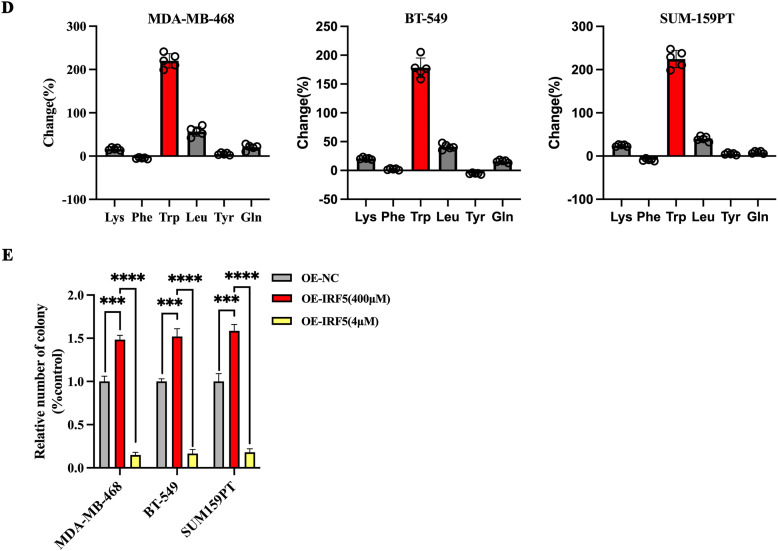
IRF5 promotes tryptophan metabolism in triple-negative breast cancer cells. (**A**) Network diagram generated by Metascape highlighting tryptophan metabolism as a key IRF5-regulated pathway involved in breast cancer metastasis. (**B**) Measurement of tryptophan levels in the culture supernatants of TNBC cells. (**C**) Quantification of kynurenine (KYN), a tryptophan metabolite, in MDA-MB-468, BT-549, and SUM159PT cells. (**D**) Assessment of amino acid (AA) consumption performed by high-performance liquid chromatography-mass spectrometry (HPLC-MS); bar graphs indicate changes relative to fresh medium. (**E**) Colony formation assays of IRF5-overexpressing TNBC cells cultured in tryptophan-rich (400 μM) and tryptophan-restricted (4 μM) media. **p < 0.01, ***p < 0.001, ****p < 0.0001

We conducted a comprehensive analysis of differential IRF5 expression in breast cancer metastatic sites, identifying 421 differentially expressed genes, including 226 upregulated and 195 downregulated genes. Subsequent network pathway clustering analysis of these genes revealed that tryptophan metabolism occupies a central role among the IRF5-associated biochemical processes, suggesting that IRF5 may influence distant metastasis of breast cancer through the tryptophan metabolic pathway (Fig. 3A). Experimental validation related to tryptophan metabolism showed that IRF5 knockdown led to a reduction of tryptophan concentration in the culture supernatants of MDA-MB-468, BT-549, and SUM159PT cells (Fig. 3B). Additionally, IRF5 downregulation significantly decreased levels of kynurenine (KYN), a tryptophan metabolite, within these breast cancer cells, further supporting the notion that IRF5 modulates breast cancer cell function via regulation of tryptophan metabolism (Fig. 3C). Consistent with these findings, amino acid consumption analysis revealed that IRF5 overexpression markedly increased tryptophan uptake in TNBC cells, while consumption of other amino acids remained largely unaffected (Fig. 3D). Moreover, culturing IRF5-overexpressing cells in tryptophan-rich medium enhanced the colony formation ability of MDA-MB-468, BT-549, and SUM159PT cells; conversely, tryptophan restriction in the culture medium reversed this enhancement (Fig. 3E).

### 3.4 IRF5 Enhances Tryptophan Metabolism in TNBC Cells by Upregulating SLC7A5 and IDO1 Expression

**Figure 4:**
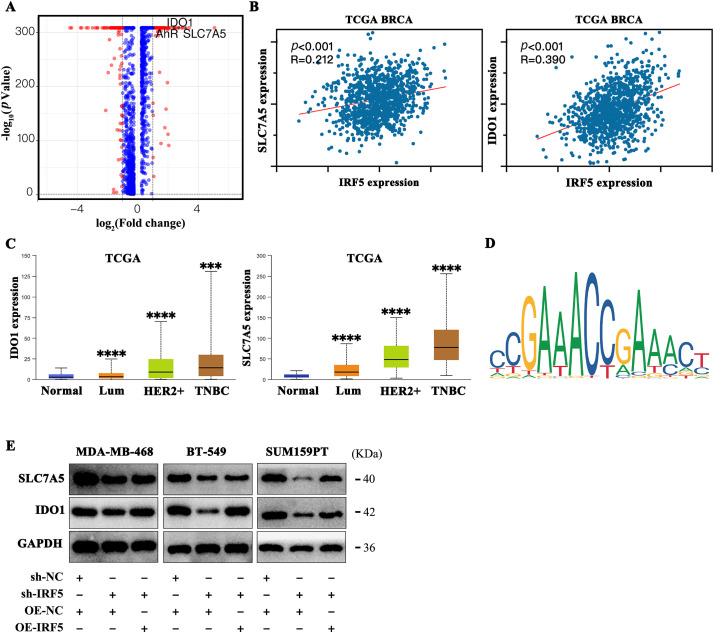
IRF5 transcriptionally upregulates SLC7A5 and IDO1 to promote tryptophan metabolism in breast cancer cells. (**A**) Volcano plot showing differential gene expression between IRF5 high- and low-expression groups, highlighting significant upregulation of SLC7A5 and IDO1. (**B**) Correlation analysis of IRF5 with SLC7A5 and IDO1 expression in the TCGA breast cancer dataset. (**C**) Expression levels of SLC7A5 and IDO1 mRNA across breast cancer molecular subtypes in the TCGA database. (**D**) Predicted IRF5 transcription factor binding motifs in the promoter regions of SLC7A5 and IDO1 based on JASPAR database analysis. (**E**) Western blot analysis showing decreased SLC7A5 and IDO1 protein levels upon IRF5 knockdown in TNBC cells, with restoration following IRF5 overexpression. ***p < 0.001, ****p < 0.0001

To clarify the molecular mechanisms by which IRF5 mediates metabolic reprogramming in triple-negative breast cancer (TNBC), we conducted differential gene ranking analysis between groups with high and low IRF5 expression. This analysis identified the tryptophan transporter SLC7A5 and the tryptophan-metabolizing enzyme IDO1 as the most significantly upregulated genes (Fig. 4A). Analysis of breast cancer data from the TCGA database revealed a strong positive correlation between IRF5 expression and that of SLC7A5 and IDO1 (Fig. 4B). Both SLC7A5 and IDO1 exhibited markedly elevated expression in breast cancer tissues, with the highest levels observed in triple-negative breast cancer subtypes (Fig. 4C). Using the JASPAR algorithm to predict transcription factor binding sites, we detected IRF5 binding motifs within the promoter regions of SLC7A5 and IDO1, suggesting that IRF5 may transcriptionally activate these genes, thereby enhancing tryptophan metabolism in breast cancer cells (Fig. 4D). Western blot quantification in MDA-MB-468, BT-549, and SUM159PT cell lines confirmed that IRF5 knockdown reduced protein levels of SLC7A5 and IDO1, whereas IRF5 overexpression restored their expression (Fig. 4E). Collectively, these results indicate that IRF5 promotes tryptophan metabolism in breast cancer cells and facilitates metastasis by transcriptionally upregulating SLC7A5 and IDO1.

### 3.5 Isoliquiritigenin Significantly Inhibits Tryptophan Metabolism and Proliferative Capacity in TNBC Cells

**Figure 5:**
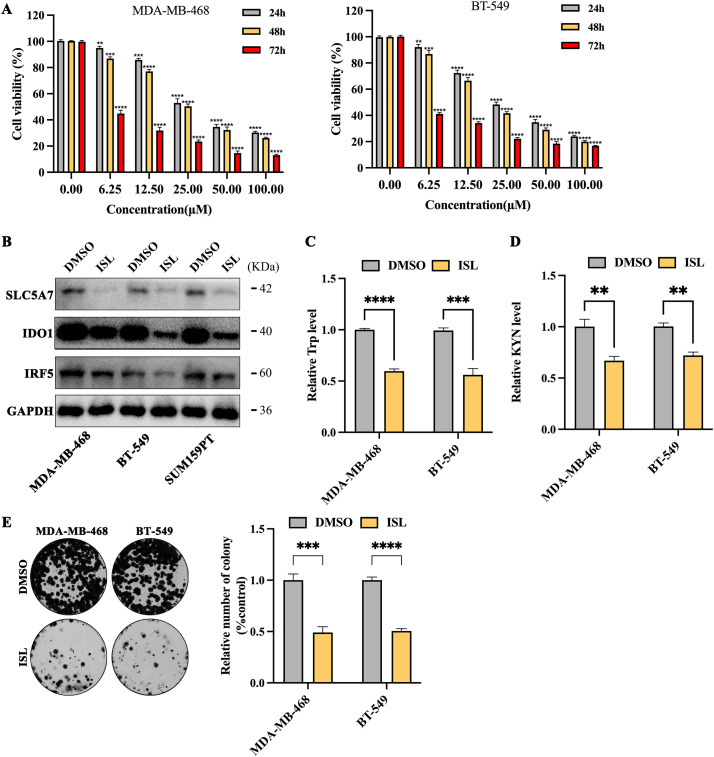
ISL suppresses tryptophan metabolism and proliferation in triple-negative breast cancer cells. (**A**) CCK-8 assays show that ISL significantly reduces cell viability of MDA-MB-468 and BT-549 cells at 24, 48, and 72 h. (**B**) ISL treatment at 30 μmol/L markedly downregulates SLC7A5 and IDO1 expression. (**C**,**D**) Quantitative analyses indicate significant decreases in tryptophan (**C**) and kynurenine (KYN) (**D**) levels in TNBC cells following ISL treatment. (**E**) Colony formation assays reveal that ISL effectively inhibits cell growth and proliferation. **p < 0.01, ***p < 0.001, ****p < 0.0001

To assess the potential clinical application of isoliquiritigenin (ISL) in treating TNBC, experiments were conducted using MDA-MB-468 and BT-549 cell lines. Both cell lines were treated with ISL (0–100 μmol/L) for 24, 48 or 72 h. The results demonstrated a significant dose- and time-dependent inhibition of cell proliferation. Specifically, the half-maximal inhibitory concentrations (IC_50_) for MDA-MB-468 cells at 24, 48, and 72 h were 35.63, 29.80L, and 4.35 μmol/L, respectively; for BT-549 cells, the corresponding IC50 values were 29.04, 22.75, and 3.01 μmol/L (Fig. 5A). Based on the 48-h IC_50_ values (29.80 μmol/L for MDA-MB-468 and 22.75 μmol/L for BT-549), a concentration of 30 μmol/L ISL was selected for subsequent experiments (Fig. 5A). Following ISL treatment, the expression levels of key downstream genes regulated by IRF5, SLC7A5 and IDO1, were markedly reduced (Fig. 5B). Further analysis of tryptophan metabolism revealed that ISL significantly decreased tryptophan levels in both MDA-MB-468 and BT-549 cells (Fig. 5C), accompanied by a notable reduction in kynurenine (KYN), a major tryptophan metabolite (Fig. 5D). These findings indicate that ISL modulates tryptophan metabolism in TNBC cells by regulating IRF5 activity. Additionally, colony formation assays demonstrated that ISL treatment substantially inhibited the growth and proliferation of MDA-MB-468 and BT-549 cells (Fig. 5E).

## 4 Discussion

Tryptophan plays a crucial role in protein synthesis, and its degradation products serve as precursors for various bioactive compounds [29]. The remodeling of tryptophan synthesis and degradation pathways has been identified as a key metabolic regulator supporting tumor cell growth [30]. Amino acid transporters are primarily localized on the plasma membrane. For instance, solute carrier family 7 member 5 (SLC7A5), a tryptophan transporter belonging to the cationic amino acid transporter, is primarily localized on the plasma membrane of tumor cells [31]. Furthermore, Shi et al. demonstrated that USP14 overexpression in colorectal cancer enhances tryptophan metabolism through the stabilization of the Indoleamine 2,3-dioxygenase 1(IDO1) protein, leading to T cell dysfunction and facilitating tumor progression [32]. Recent studies have shown that numerous candidate drugs targeting key points in the tryptophan metabolic pathway have entered clinical trials [33]. However, despite the high therapeutic potential of this approach, clinical efficacy remains limited due to challenges including strong drug resistance and significant side effects.

Our mechanistic investigations reveal that IRF5 directly regulates tryptophan metabolism by upregulating the expression of SLC7A5, a critical amino acid transporter, and IDO1, a key enzyme mediating tryptophan catabolism [31,34]. The tryptophan metabolic pathway is increasingly recognized as a central axis in cancer immune evasion [15,35,36]. IDO1-mediated degradation of tryptophan leads to local depletion of this essential amino acid and accumulation of immunosuppressive metabolites such as kynurenine, which dampen effector T cell function and promote regulatory T cell expansion [32]. SLC7A5 facilitates the influx of large neutral amino acids, including tryptophan, thereby sustaining the metabolic demands of proliferating tumor cells and further feeding immunosuppressive metabolism. Besides, Ma et al. reported that circARID1A promotes gastric cancer growth by stabilizing SLC7A5 mRNA through forming a complex with IGF2BP3. This enhances SLC7A5 expression and activates the AKT/mTOR pathway, suggesting the circARID1A-IGF2BP3-SLC7A5 axis as a potential therapeutic target [37]. Our findings underscore the importance of the IRF5-SLC7A5-IDO1 axis as a regulatory node linking metabolic reprogramming with immune modulation in TNBC.

Notably, the immunomodulatory effects of ISL may extend beyond direct tumor cell metabolism [38]. IRF5 is a key transcription factor in immune cells, particularly macrophages and dendritic cells, involved in shaping the inflammatory milieu [19,20,39]. Although our study primarily focuses on tumor-intrinsic IRF5 functions, future investigations should explore ISL’s impact on the immune compartment of the TME, potentially enhancing antitumor immunity through immune cells. Such immune-boosting properties could synergize with metabolic inhibition, positioning ISL as a multifaceted therapeutic candidate.

This study, while providing novel insights into the anti-tumor mechanisms of ISL in TNBC, has several notable limitations. First, it predominantly relies on *in vitro* experiments using TNBC cell lines. The absence of *in vivo* animal models represents a critical gap, as *in vitro* results may not fully recapitulate the tumor microenvironment, immune interactions, or physiological cancer progression observed in living organisms. Second, although our study established an association between ISL, IRF5, and tryptophan metabolism in TNBC cells, the precise molecular mechanisms through which ISL regulates IRF5 remain undefined. Addressing these limitations will provide a more solid foundation for developing ISL as a potential therapeutic strategy for TNBC. In summary, our study identifies IRF5 as a novel driver of TNBC progression through its regulation of tryptophan metabolism via SLC7A5 and IDO1. ISL effectively inhibits this pathway, suppressing tumor growth and metabolic reprogramming. These insights expand the understanding of TNBC pathobiology and highlight the therapeutic potential of targeting metabolic-immune crosstalk. Further preclinical and clinical studies are warranted to validate these findings and explore combinatorial approaches that harness the metabolic and immunological vulnerabilities of TNBC.

## Data Availability

The datasets and materials supporting the findings of this study are available from the corresponding authors upon reasonable request.
